# Efficient quantization of painting images by relevant colors

**DOI:** 10.1038/s41598-023-29380-8

**Published:** 2023-02-21

**Authors:** Zeinab Tirandaz, David H. Foster, Javier Romero, Juan Luis Nieves

**Affiliations:** 1 Department of Electrical and Electronic Engineering, University of Manchester, Manchester, M13 9PL UK; 2 Department of Optics, University of Granada, 18071 Granada, Spain

**Keywords:** Human behaviour, Computational science, Computer science, Computational biology and bioinformatics, Image processing, Machine learning, Electrical and electronic engineering, Sensory processing, Neuroscience, Colour vision, Object vision, Pattern vision

## Abstract

Realistic images often contain complex variations in color, which can make economical descriptions difficult. Yet human observers can readily reduce the number of colors in paintings to a small proportion they judge as relevant. These relevant colors provide a way to simplify images by effectively quantizing them. The aim here was to estimate the information captured by this process and to compare it with algorithmic estimates of the maximum information possible by colorimetric and general optimization methods. The images tested were of 20 conventionally representational paintings. Information was quantified by Shannon’s mutual information. It was found that the estimated mutual information in observers’ choices reached about 90% of the algorithmic maxima. For comparison, JPEG compression delivered somewhat less. Observers seem to be efficient at effectively quantizing colored images, an ability that may have applications in the real world.

## Introduction

Images of the real world and of the objects within it reveal the spatial and spectral complexity of natural surfaces^1^ and their illumination^2,3^. But the impression of chromatic detail is likely to be founded on partial information, limited by our peripheral color awareness^4^, pattern of gaze^5^, attention^6^, memory^7^, and the emotional and semantic content of the scene^8,9^, and, more fundamentally, by the relative abundances or frequencies of the different colors present^10,11^. Of course, not all the colors in a scene are needed in order to describe or remember it, and different methods have been used to estimate which colors are relevant.

The most direct approach is to ask human observers to make the required judgments. In a psychophysical experiment^12^, observers selected those pixels in an image of a painting they considered to belong to a “relevant chromatic area”. The images were of 20 paintings in the Prado Museum^13^, Madrid, and of 20 artworks from the database of Khan et al.^14^. Observers could choose as many colors as they wished. This procedure yielded a mean of 21 relevant colors for each image, with the number and identity of the colors varying with both the image and the observer.

An alternative theoretical approach is to use colorimetric methods^15^. In such an analysis^16^, the approximately uniform color space CIELAB^17^ was divided into cubic cells whose side length was a multiple of the smallest discriminable step, and colorimetric arguments were then used to assign the colors of a scene to those cells. The images were of 4266 artworks from the database of Khan et al.^14^. The analysis yielded a mean of 18 relevant colors per image, the number and identity again varying with the image. Further details of this colorimetric method and of observers’ experimental judgments are given in Methods.

Whatever the method, the use of relevant colors offers a way to simplify an image by quantizing it, that is, by reducing a large, essentially continuous range of colors to a much smaller discrete set. It is unclear, however, whether this process is efficient. Does it capture the most information about the image for a given number of relevant colors?

The present analysis addresses this question. Information was quantified by Shannon’s mutual information^18^, though other formulations are possible^19^. Estimates were made of the mutual information between images and the quantized representations implied from observers’ relevant colors and from the colorimetric method and then compared with estimates of the maximum mutual information by general optimization methods. These methods used clustering to partition the set of colors in each image into *n* subsets according to some cost or objective function. Although values of *n* were matched to the number of relevant colors for each image obtained by each observer and by the colorimetric method, methods for objectively determining *n* were also considered. In all, five clustering algorithms were considered, and were based on *k-*means++^20^, maximum entropy clustering (MEC)^21,22^, expectation maximization with a Gaussian mixture model (GMM)^21,23^, minimum conditional entropy (minCEntropy)^24–26^, and Graph-Cut^27–30^. Each is explained in Methods, but minCEntropy has the notional advantage that it is designed to maximize the mutual information between the data and the clustering.

**Figure 1:**
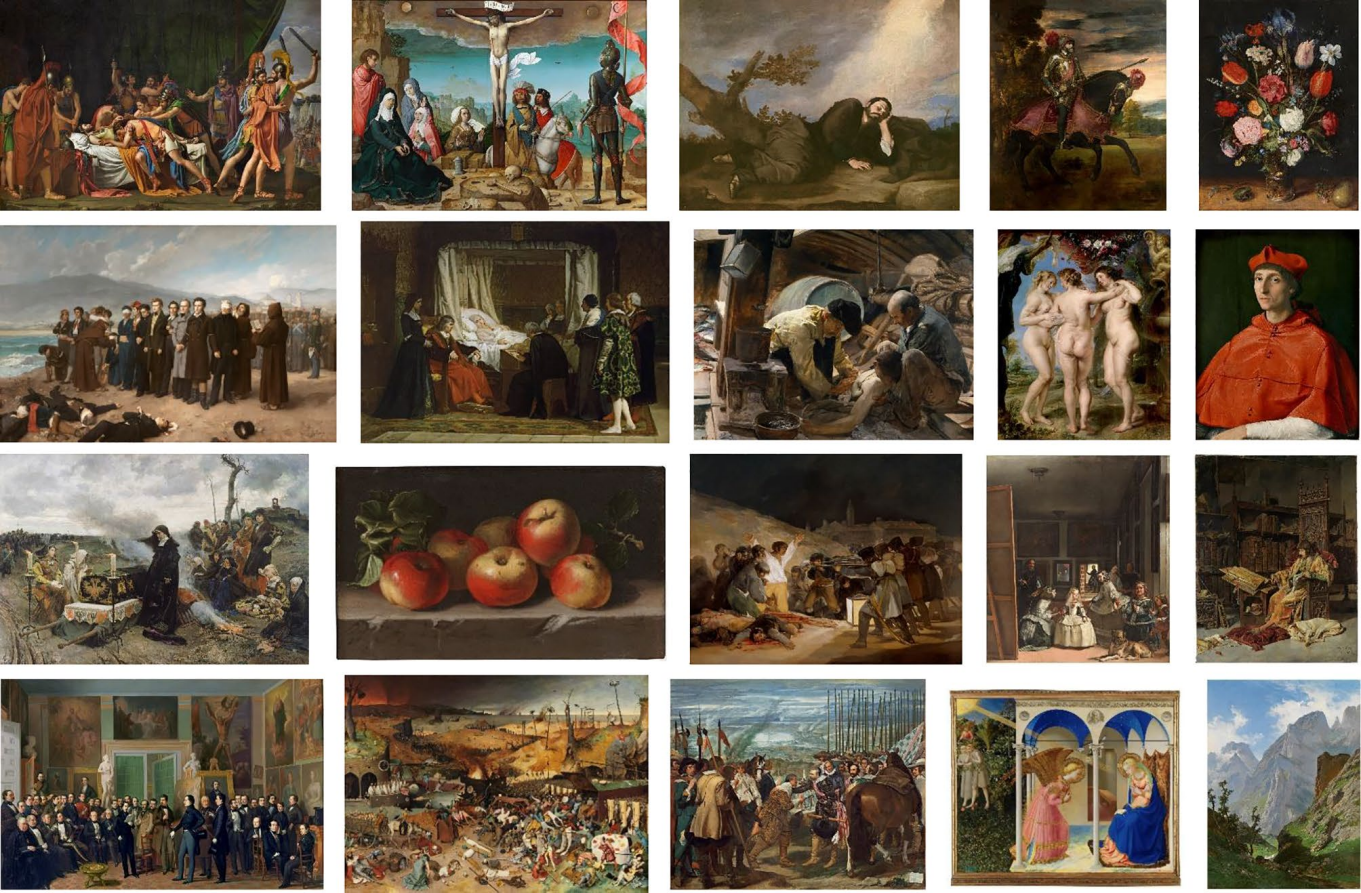
Montage of images of conventionally representational paintings in the Prado Museum^13^, adapted from Ref.^12^, Fig. 1, licensed under CC BY 4.0. The color gamuts of each of these images are illustrated in Fig. 4 in Methods.

The test images are illustrated in Fig. 1. They were chosen for their complex spatio-chromatic content and realistic character and are a subset of conventionally representational painting images used earlier^16^, for which observer estimates of relevant colors were already available^12^. The color gamuts of each of these images are illustrated in the Methods section. An example of a quantized representation is shown later. Other examples are available elsewhere^12,16^.

For clarity, this analysis should be distinguished from more abstract approaches^31^ in which the efficiency of color naming itself is evaluated with uniform color palettes such as the Munsell set^32^. It should also be distinguished from color categorization with a fixed set of basic or salient color terms^33^, and from image classification with a set of color descriptors for database retrieval^34^. The relevant colors used here were distributed non-uniformly in each image, they were not drawn from fixed categories, and observers were instructed not to name them.

It was found that the estimated mutual information in observer judgments of relevant colors was close to the estimates from all five clustering methods, including minCEntropy, and the colorimetric method. Observers seem to be efficient at effectively quantizing colored images of paintings.

## Results

### Efficiencies of representations by relevant colors

**Figure 2:**
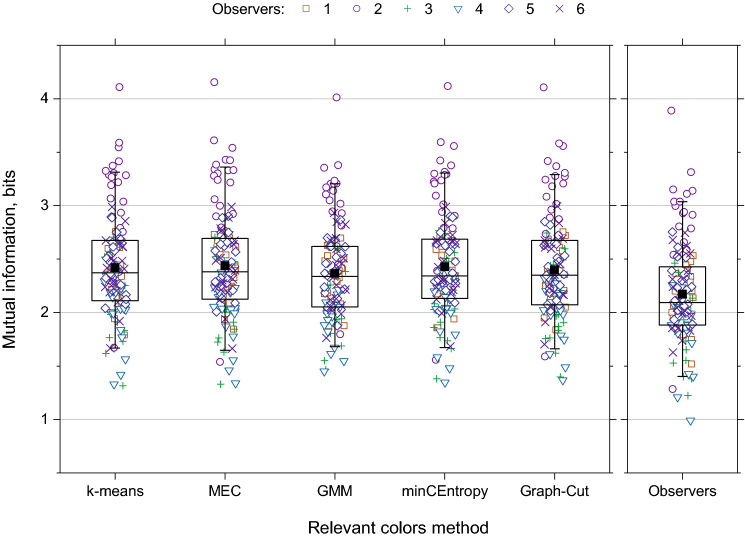
Information from relevant colors. Estimated mutual information between each of the 20 images in Fig. 1 and their quantized representations by relevant colors is shown for five clustering methods and six human observers, indicated by different symbols. Horizontal jitter has been added to reduce overlap between symbols^35^. Estimates for individual images are not distinguished. Boxplots represent median (center line), mean (solid square), interquartile range (rectangle), and the 5th and 95th percentiles (whiskers).

Figure 2 shows the estimated mutual information between each image in Fig. 1 and its quantized representation by relevant colors for each of the five clustering methods, *k*-means++, MEC, GMM, minCEntropy, and Graph-Cut, and by individual observers. The 120 data points for each method derive from the 20 images and the six observer’s choices of numbers of relevant colors for each image^12^. The data are summarized by the superimposed boxplots.

The five clustering methods produced closely similar mean levels of estimated mutual information of about 2.4 bits, and observers slightly lower levels, about 2.2 bits. The variance in the plotted data, however, is potentially misleading in that the estimated mutual information for an image and the number of relevant colors *n* covary across methods and observers. This confound was circumvented by calculating the efficiency of one method relative to another for each image and for the same number of relevant colors *n*. Values of *n* were drawn from observers’ choices, except for the colorimetric method, which determined *n* automatically for each image. That is, for *k*-means++, MEC, GMM, minCEntropy, and Graph-Cut, multiple values of *n* were available from observers for each image whereas, for the colorimetric method, only one value was available for each image.

The choice of reference method is not critical, and the minCEntropy method was chosen for the property mentioned earlier, that is, maximizing the mutual information between the data and the clustering. Thus, suppose that *I* is the mutual information between an image and its quantized representation by relevant colors estimated by a particular method and that *I*_ref_ is the corresponding quantity estimated by the minCEntropy method. Then the efficiency $\eta$ of the method for this image and value of *n* is

$$\eta = \frac{I}{{I_{{{\text{ref}}}} }},$$

which can be averaged over all images and all *n*. Table 1 shows mean efficiencies $\eta$ calculated with respect to the minCEntropy estimates, along with 95% confidence limits.

**Table 1:** Efficiencies of methods for estimating information from relevant colors relative to minCEntropy clustering.

Estimation method	Mean efficiency $\eta$ (%)
*k-*means++	99.7 (99.3, 100.0)
MEC	100.3 (100.0, 100.7)
GMM	97.8 (96.9, 98.8)
Graph-cut	98.8 (98.5, 99.2)
Colorimetric	81.4 (78.0, 83.6)
Human observers	89.4 (88.1, 90.6)

Mean efficiencies $\eta$ are given for three clustering methods, the colorimetric method, and six human observers. Values of $\eta$ were averaged over 20 images and numbers of relevant colors *n*. Estimated 95% BCa confidence limits^36^ are shown in parentheses, based on 1000 bootstrap replications.

Estimates by observers are close to but less than the optimum method, with a mean efficiency of 89%. In the light of the 95% confidence limits, this difference is reliable. Estimates with the other clustering methods did not differ reliably from the minCEntropy estimate.

### Variation in numbers of relevant colors

Observers were free to choose as many relevant colors as they wished^12^, and, as indicated earlier, they chose different numbers *n* with each scene. This is evident in Fig. 2 and was confirmed statistically (Kruskall-Wallis test, *p* < 0.001). Crucially, though, observers’ efficiencies $\eta$ in making those choices did not differ significantly (Kruskall–Wallis test, *p* = 0.8).

With different numbers of relevant colors for each image, does mutual information increase predictably as *n* increases?

**Figure 3:**
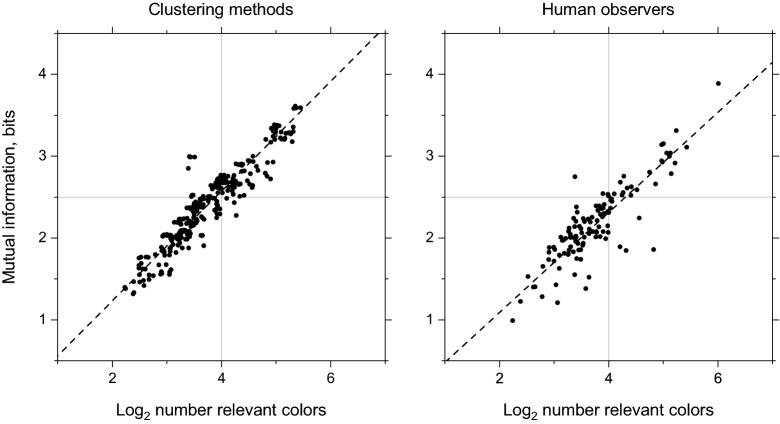
Information and number of relevant colors. Estimated mutual information is plotted against logarithm to the base 2 of the number of relevant colors *n* for the five clustering methods and six human observers. Horizontal jitter has been added to reduce overlap between symbols^35^. The dashed lines represent linear regressions on the unjittered data.

Figure 3 shows estimated mutual information plotted against the logarithm to the base 2 of the number of relevant colors for the five clustering methods and six human observers. Both the clustering methods and observers behaved broadly appropriately, that is, as the number of relevant colors increased, so did the estimated mutual information. The slopes of the regression fits were similar (95% confidence limits in parentheses): 0.67 (0.65, 0.69) and 0.61 (0.53, 0.66) for the clustering methods and observers, respectively.

### Objective estimates of numbers of relevant colors

Rather than depend on observer estimates of the number of relevant colors *n*, is there an objective way of deciding? To this end, three algorithmic methods for estimating *n* for each image were tested. These were the Caliński-Harabasz^37^ method with upper limits of *n* = 40 and *n* = 80; the Davies–Bouldin^38^ method with upper limit *n* = 80; and an adaptive *k*-means method^39^, which does not require initialization. All are described more fully in Methods.

Table 2 shows the optimum numbers of clusters averaged over the 20 images, along with corresponding averages for observers.

**Table 2:** Estimated optimum numbers of clusters, averaged over 20 images, according to three methods, and different initial values.

Caliński-Harabasz, upper limit 40	Caliński-Harabasz, upper limit 80	Davies-Bouldin, upper limit 80	Adaptive *k*-means	Human observers
8.2 (6.2, 11.4)	8.9 (6.5, 12.6)	2.5 (2.2, 3.1)	4.0 (3.6, 4.4)	15.4 (14.0, 17.1)

Data for human observers are included for comparison. Estimated 95% BCa confidence limits^36^ are shown in parentheses, based on 1000 bootstrap replications.

The problems of consistency are evident. The three methods deliver mean optimum numbers that differ reliably from each other, and which fall reliably below those selected by human observers.

### Comparison with JPEG coding

The quantized representation of an image by relevant colors can be thought of as a lossy compression. As a demonstration, mutual information estimates by the minCEntropy and colorimetric methods and from observers were compared with those from the popular JPEG compression algorithm^40^, although accurate color quantization is not its primary goal. The JPEG implementation in MATLAB (version 9.10.0.1602886 (R2021a), The MathWorks, Inc., Natick, MA) was used with a quality setting of zero to minimize the normally large number of colors in the representation (e.g., for the image in the second row, third column of Fig. 1, a quality setting of 5 gave 6040 colors and a setting of zero gave 2611 colors).

Table 3 shows estimates of mutual information and numbers of colors *n* with three of the images of Fig. 1 that produced the largest visual differences across methods. Unlike Table 1 where each method was compared with the minCEntropy method for the same *n*, this normalization was not possible here. Since the colorimetric method determined *n* automatically for each image, this value was used for the minCEntropy method. The observer representation was then chosen as the one with the value of *n* closest to that of the colorimetric method.

**Table 3:**
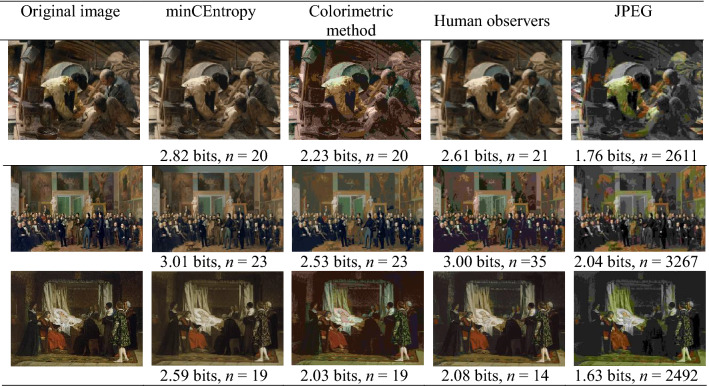
Quantized representations of an image by relevant colors and JPEG compression.

The original image from Fig. 1 is shown in the first column. Subsequent columns show the image quantized by relevant colors estimated by the minCEntropy method, the colorimetric method, a human observer, and JPEG with the fewest colors. The estimated mutual information in bits and number of relevant colors *n* are shown beneath the corresponding image.

Even with a JPEG quality setting of zero, the mean number of JPEG unique colors was 3.9 × 10^3^, more than two orders of magnitude larger than with the other three methods. Yet the minCEntropy method, the colorimetric method, and individual observers were able to preserve 2.6 bits of information on average over all 20 images, more than the 2.1 bits preserved on average with JPEG. For the calculation of the efficiency of JPEG relative to the minCEntropy method, it was impracticable to use the same *n* with minCEntropy, and the value of* n* from the colorimetric method was used instead. The mean JPEG efficiency (with 95% confidence limits) was 72.3% (68.6%, 76.1%).

The effects of information loss on appearance are obvious. For all three images in Table 3, JPEG rendered light brown as light green, most noticeably the shirt of the figure in the top row on the left, presumably a side-effect of downscaling color in JPEG. The colorimetric method showed a similar but smaller bias. Both the minCEntropy method and individual observers rendered all the colors well.

### Color rendering

To extend the comparisons of color rendering across all the images, mean color differences were estimated between each painting image and its quantized representation. The RGB values at each pixel were converted to CIE 1931 XYZ tristimulus value values (2° observer) and color differences evaluated in the approximately uniform color space CIECAM02-UCS^17^ with respect to a 6500 K illuminant. These differences were then averaged over the whole image. For comparison, color differences were also evaluated in the somewhat less uniform color space S-CIELAB^41,42^, which takes into account the spatial-frequency filtering of the whole image by the eye. The same illuminant was assumed. Table 4 shows these color differences averaged over all 20 images for both color spaces. As with Table 3, *n* was set by the colorimetric method.

**Table 4:** Color rendering by relevant colors in two color spaces^17,41,42^.

Estimation method	Mean CIECAM02-UCS color difference	Mean S-CIELAB color difference
minCEntropy	3.7 (3.5, 4.0)	4.1 (3.9, 4.4)
Colorimetric method	7.9 (7.7, 8.2)	9.0 (8.9, 9.3)
Human observers	5.5 (5.1, 6.0)	6.2 (5.7, 6.6)
JPEG	9.1 (8.6, 9.5)	12.7 (12.0, 13.3)

Color differences between the original image and its quantized representation are shown for minCEntropy clustering, the colorimetric method, six human observers, and JPEG, with values averaged over the 20 images. Estimated 95% BCa confidence limits^36^ are shown in parentheses based on 1000 bootstrap replications.

In principle, a just perceptible color difference of about 0.5 in CIECAM02-UCS is around half that value in CIELAB space^43^, but larger values of about 1.5 in CIECAM02-UCS and 2.2 in CIELAB space have also been used with whole images of natural scenes^44^. With any of these thresholds, the color differences in Table 4 manifestly represent detectable effects in CIECAM02-UCS and CIELAB space.

That there should be detectable differences is not unreasonable. Quantization by relevant colors entails a reduction in the number of unique image colors of about 5000 to 1, on average. While many of the original colors coincided with their quantized values, there were inevitably many other colors that did not.

## Discussion

Representing images in terms of a limited number of relevant colors reduces their spatial and spectral complexity, but with an inevitable loss of information. The present analysis showed that with representational painting images, the estimated information captured by human observers in their choices of relevant colors^12^ reached about 90% of that possible by algorithmic clustering methods, all of which maximized the estimated mutual information between the image and the clustering. Observers seem to be efficient at effectively quantizing these images.

In making their choices, observers were not limited to any particular number of relevant colors^12^. Reassuringly, whether observers settled on a few or many relevant colors (numbers ranged from 5 to 68 across scenes), the colors they chose approached the optimum in each case. As Fig. 3 shows, mutual information estimates increased approximately linearly with the logarithm of the number of colors, though not quite as rapidly as with algorithmic clustering methods. With this variation, it seems likely that establishing an objective estimate of an optimum number of relevant colors for a given image will require explicit observer modeling, along with additional constraints. As was clear from Table 2 algorithmic clustering methods produced estimates of optimum numbers that were all much lower than for observers.

The focus of this analysis has been on information processing, not on color rendering per se. Even so, there is a parallel in the ordering of the outcomes in the two approaches for a given number of colors. Thus, in Table 1, the mutual information estimates between original and quantized images progressively decreased across clustering methods, observers, and the colorimetric method. Conversely, in Table 4, the mean color differences between original and quantized images progressively increased across the same range. But the two kinds of measure are not equivalent. The key distinction is that mutual information depends on the relative frequencies of image colors whereas color differences depend on the metrical properties of the space used to represent colors. Mutual information is not necessarily maximized by minimizing color differences^45,46^.

There are several caveats to this analysis. First, in practice, the relevant colors characterizing an image need not be the same as the set of colors significant for a particular task. Some colors may additionally have a subjective salience^47–49^, affecting measures such as gaze direction^5^ and search performance^50,51^. Second, although the five clustering methods invoked different criteria to achieve a solution, the possibility remains that other methods could yield higher estimates. Third, the comparison with quantization by JPEG was informative but intended only for illustration, since JPEG is designed for overall image compression, not efficient color quantization. A range of alternative color spaces has been considered for JPEG^52^, though an adaptable color coding using relevant colors may offer still better performance^53,54^. Fourth and last, all the information estimates were based on images of conventionally representational paintings rather than on images of actual scenes, objects, or individuals. That said, paintings and real scenes are known to have overlapping gamuts, elongated in the yellow-blue direction, albeit with the gamuts of paintings tilted in the red direction^55–57^.

Given the levels of performance estimated here, if observers are equally efficient in judging relevant colors in the real world, the resulting quantized representations may find applications in a variety of tasks, including the description and memorization of scenes, and their eventual recall.

## Methods

### Image data

The 20 test images of conventionally representational paintings from the Trecento to the Romantic era were the same as those used in a psychophysical study^12^ to obtain observer estimates of relevant colors. They represent rural landscapes, indoor scenes, still lifes, portraits, and historical events. The images, made available by the Prado Museum^13^, were coded as 24-bit RGB with 6,520,320 to 9,682,560 pixels per image. The mean number of unique colors in each image was 1.3 × 10^5^ within the bit resolution of the dataset.

**Figure 4:**
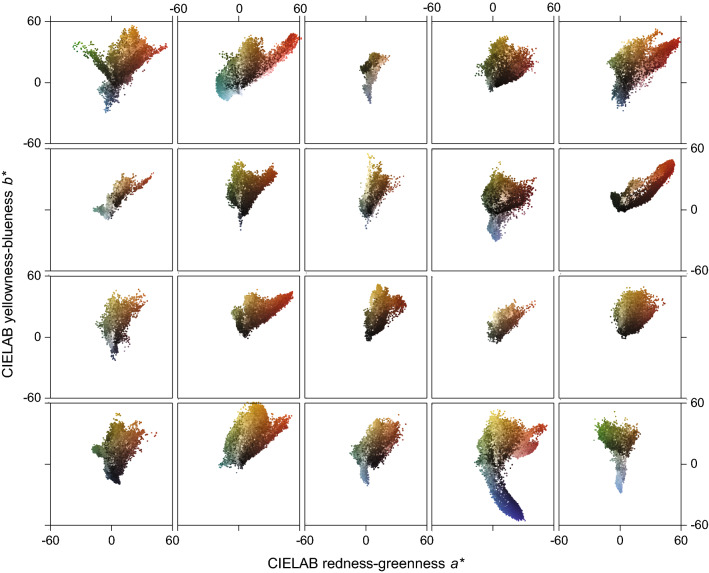
Color gamuts of painting images in Fig. 1. Each plot shows the CIELAB^17^ chromaticity coordinates (a^*^, b^*^) of pixels drawn uniformly from the corresponding image in Fig. 1, which has the same row-column layout. Only about 1% of pixels are plotted to more clearly reveal density variations.

Figure 4 illustrates the gamuts of the colors in each of the painting images in Fig. 1. Pixels were drawn uniformly from each image and plotted, for consistency with earlier data, in the chromaticity plane of the approximately uniform color space CIELAB^17^, where a^*^ correlates with redness–greenness and b^*^ with yellowness–blueness. Notice the small gamut for the painting in the top row, column 3 of Fig. 1 and the extended blue lobe for the painting in the bottom row, column 4 of Fig. 1. The locations of relevant colors with these image gamuts are shown in Ref.^12^.

### Observer data

Data on relevant colors chosen by human observers were taken from a previously reported psychophysical experiment^12^ with six participants, three men and three women. The number of observers was of the same order as in similar works^58–60^ and the homogeneity of their responses is considered in the Results section. The observers sat in front of a monitor with head position stabilized by a chinrest. Images of paintings were displayed with PsychToolbox-3^61^, a package consisting of MATLAB and GNU Octave functions and Python toolkits that provided an interactive visual environment. Observers used a mouse to select within the image representative locations they considered “valid instances of relevant colors”. The recorded relevant colors were defined by the average of 25 pixels around each of the selected locations. The quantized images were created after the experiment and so did not influence observers’ choices. The study was conducted according to the guidelines of the Declaration of Helsinki and approved by the Institutional Review Board (Ethics Committee) of the University of Granada, Spain (protocol code 1746/CEIH/2020). Informed consent was obtained from all participants in that study. Additional detail about the choice of observers is available in Ref.^12^.

### Algorithmic clustering methods

The colorimetric method used here^16^ mapped RGB pixel values of the original image into CIELAB color space^17^, which was then divided into small cubic cells with fixed sides. Based on the number of pixels in each cube and whether their luminance and chroma values were within a certain range, each cube was designated as relevant or not. Pixels with values in the same relevant cell were assigned the average for that cell, and pixels in non-relevant cells were assigned the closest relevant cell average.

The MEC method^21,22^ incorporates Shannon entropy^18^ in the objective function. Initial clusters were obtained by *k*-means++, and the entropy-based objective function was then maximized, equivalent to minimizing negative entropy. Clusters were updated iteratively until the objective function value converged.

With the GMM method^21,23^, pixel values are assumed to be generated by *k* Gaussian mixture components. The built-in MATLAB *k*-means++ clustering function kmeans was used to obtain an initial clustering with the mean, covariance, and membership weights forming the objective function. Iterative expectation maximization was applied until there was no significant change in the objective function value.

The MinCEntropy method^24–26^ is a hill-climbing method. It maximized the mutual information between the original pixel values and their quantized representation by clustering. Initialization was again by *k*-means++ and clustering was updated iteratively until the clusters stabilized.

The Graph-Cut method ^27–30^ is an energy-minimization method based on graph cuts. Initialization was by *k*-means. The graph was then constructed with pixels as its nodes and the probability of pixels belonging to the same cluster as its weighted edges. The algorithm then cut through the weak edges to yield a final clustering.

### Algorithmic estimates of cluster numbers

Three algorithms for estimating the number of clusters in each image were evaluated and compared with results from human observers. The Caliński-Harabasz index^37^, also known as the variance ratio criterion (VRC), determines the ratio of the sum of between-clusters dispersion and inter-cluster dispersion for all clusters, where the dispersion is the sum of squared distances. A range was set for the number of clusters and the number within the range with the highest VRC chosen as the optimum number of clusters. The Davies-Bouldin index^38^ is based on the ratio of within-cluster distances to between-cluster distances. The measure is unrelated to VRC. The index values were measured for a range of numbers of clusters, and the number with the lowest Davies-Bouldin index was selected as the best solution. Adaptive *k*-means clustering^39^ partitions the pixel values without a predetermined number of clusters and is different from the built-in *k*-means++ mentioned elsewhere. The clusters were initialized with the mean values of the RGB image planes and then updated with the Euclidean distances of pixel values from these centers until they stabilized.

### Estimating mutual information

For a pixel chosen randomly from an image, its RGB values **a** = (*R*, *G*, *B*) may be treated as an instance of a trivariate discrete random variable **A** whose probability mass function (pmf) is *p* say. The entropy *H* (**A**) of **A** is defined^18^ by

$$H({\mathbf{A}}) = - \sum\nolimits_{{\mathbf{a}}} {p({\mathbf{a}})} \log p({\mathbf{a}})$$

where **a** ranges over the gamut of pixel values and *H* (**A**) is in bits if, as here, the logarithm is to the base 2. For two images represented by random variables **A**_1_ and **A**_2_ with respective pmfs *p*_1_ and *p*_2_, the mutual information *I* (**A**_1_; **A**_2_) between **A**_1_ and **A**_2 _can be defined as

$$I\left( {{\mathbf{A}}_{{1}} ;{\mathbf{A}}_{{2}} } \right) = H\left( {{\mathbf{A}}_{{1}} } \right) + H\left( {{\mathbf{A}}_{2} } \right) - H\left( {{\mathbf{A}}_{{1}} ,{\mathbf{A}}_{{2}} } \right),$$

where *H* (**A**_1_, **A**_2_) is the entropy of **A**_1_ and **A**_2_ taken jointly^18^. Naïve estimates of $p$ can be obtained by binning the space of color values **a** into a finite number of cells and counting the frequency of occurrences in each cell. This procedure can, though, lead to bias when the number of samples is small^62^. As elsewhere^63^, a bias-corrected estimator $\hat{H}_{{\text{G}}} ({\mathbf{A}})$ due to Grassberger^64^ was used instead. Estimates were made of the mutual information *I* (**A**; **A**_q_) between random variables **A** and **A**_*q*_ representing, respectively, an original RGB image and its quantized representation by relevant colors.

## Data Availability

The images of paintings analysed in this study are available at https://www.museodelprado.es/en/the-collection. Software for estimating mutual information is available at https://github.com/imarinfr/klo. Software for implementing the clustering methods is available at https://github.com/kailugaji/Color_Image_Segmentation, https://github.com/shaibagon/GCMex, and https://github.com/ZT-HT/Clustering_minCEntropy. Software for converting images to S-CIELAB color space is available at https://github.com/wandell/SCIELAB-1996/.
